# The modulatory effect of oxygen -releasing gel versus 0.8% hyaluronic acid gel incorporated hydroxyapatite bone graft in the management of grade II mandibular furcation defects: a randomized clinical control trial

**DOI:** 10.1590/1678-7765-2026-0115

**Published:** 2026-08-21

**Authors:** Pavitra Jayakumar, Sharmistha Vijay, Debopriya Chatterjee, Setu Mathur

**Affiliations:** 1 Autonomous researcher Jaipur India Autonomous researcher, Jaipur, India; 2 Government Dental College and Hospital Jaipur Rajasthan University of Health Sciences Department of Periodontics Jaipur India Government Dental College and Hospital Jaipur, Rajasthan University of Health Sciences, Department of Periodontics, Jaipur, India

**Keywords:** Open flap debridement, Oxygen-releasing gel, Hyaluronic acid gel, Hydroxyapatite bone graft

## Abstract

**Aim:**

The intricate architecture of multirooted teeth challenges access to the furcation for treatment. This study aimed to assess the clinical and radiographic efficacy of oxygen-releasing oral gel and 0.8% hyaluronic acid gel along with hydroxyapatite bone graft to treat Class II furcation defects.

**Methodology:**

Overall, 45 (one site per participant, aged from 20 to 60 years) mandibular molars with periodontitis (stage II and stage III, grade A and B) and class II buccal furcation defects were treated in this study. Selected sites were randomly divided into three groups. In group 1, placebo gel was placed after open flap debridement (OFD), in group 2- 0.8% hyaluronic acid gel + hydroxyapatite bone graft was placed after OFD, and, in group 3, oxygen-releasing oral gel + hydroxyapatite bone graft was placed after furcation defect OFD. Clinical parameters such as gingival index, plaque index, relative vertical clinical attachment level, relative horizontal clinical attachment level, and probing pocket depth were recorded at baseline and six months later. Radiographic measurements—intra bony defect & % bone fill—were measured and calculated by cone beam computed tomography. Results: The mean reduction in intra bony defect was highest in Group 3 (1.433±0.594), followed by Groups 2 (1.000 ± 0.000) and 1 (0.700±0.414). Group 1 showed the lowest mean bone fill (34.69%±12.14), which was significantly lower than in Groups 2 (46.87%±4.71) and 3 (50.93%±4.62), with p-values of (p<0.001) in both comparisons.

**Conclusion:**

Within the limitations of this study, its results imply that oxygen-releasing gel along with hydroxyapatite bone graft in surgical periodontal therapy to treat class II furcation defects has shown superior regenerative potential. Clinical Trial Register: CTRI/2025/02/081311

## Introduction

Periodontitis is a multifactorial inflammatory infection that causes loss of connective tissue and alveolar bone around teeth.^1^Dental biofilm causes dysbiosis, leading to periodontitis, which is characterized by a disbalance between the host reaction and the microbial biofilm that gradually destroys the periodontium.^2,3^

The immune system is a double-edged sword as it protects and destroys the periodontium by triggering an inflammatory reaction in response to microbial attacks.^4^

The anatomical region of a multirooted tooth in which the roots diverge is known as the furcation. The pathologic resorption of the supporting alveolar bone within a furcation usually results from advancing aggressive or chronic periodontitis, causing furcation invasion or involvement.^5^The degree of furcation involvement determines how severe attachment loss is in teeth with many roots.^6^

Mandibular class II furcation involvements challenge periodontal therapy because of their multi-rooted tooth morphology and higher risk of molar tooth loss during chronic periodontitis in comparison to single-rooted teeth.^7^The intricate structure, limited entrance, and variable morphological ridges, convexities, and concavities of furcation defects make instrumentation more challenging.^7,8^

The distinct structural features of furcation areas restrict the accessibility of instruments and result in a less-than-ideal response to traditional periodontal treatments. Hence, additional therapy is necessary.^9^

Numerous bone graft materials have been utilized and evaluated in clinical settings, including intraoral and extraoral autografts, allografts, xenografts, and synthetic/semi-synthetic materials such as bioactive glass, tricalcium phosphate, and hydroxyapatite (HAP).^10^

When used to treat periodontal furcation deficits, HAP has produced clinically satisfactory outcomes as it enables osteogenic cells to multiply on the surfaces of the existing bone as it acts as a scaffold. HAP showed enhanced regeneration potential when combined with biologically active material.^11,12^HAP is an alloplastic material. Although the clinical result of using alloplastic grafts for periodontal bone regeneration seems promising, histologically, these grafts get enclosed by connective tissue with little or no bone formation;^13^ hence, an adjunct use of a local drug delivery agent can increase its regenerative potential.

Lactoferrin and active oxygen, two powerful compounds that complement one another, can be used as a local drug delivery agent to aid the reduction of anaerobic bacteria, making it unique.^14^

It has anti-inflammatory properties, supporting the early inflammatory stages of tissue healing such as granulation tissue formation and synthesis of extracellular matrix components such as collagen and hyaluronan and stimulating fibroblast and keratinocyte migration and proliferation.^15,16^

Oxygen-releasing gel has antibacterial, antihistamine, and antimycotic properties, neovascularization, toxin clearance, new blood cell promotion and production, and stem cell synthesis boost, accelerating healing.^17^

Combination therapy has been used in this study: hyaluronic acid (HA) along with HAP. HA encourages cell survival, proliferation, and early osteogenic differentiation. It accelerates cell migration, differentiation, and the healing process. Because of its anabolic properties, it plays an important role in non-surgical periodontal therapy, peri-implantitis management, root covering procedures, furcation involvement treatment, and socket preservation.^18,19^ Combination therapy simultaneously uses two or more periodontal reconstructive techniques or materials to enhance regenerative outcomes. It aims to achieve an additive or synergistic effect by combining biological and mechanical principles involved in tissue regeneration.^20^

Hence, this study aimed to assess the clinical and radiographic efficacy of oxygen-releasing oral gel and 0.8% HA gel along with hydroxyapatite bone graft in the treatment of mandibular class II furcation defects.

## Methodology

This is a randomized controlled clinical, single-centric study in which 45 male and female participants with periodontitis (stage II and stage III, grade A and B) who were aged from 20 to 60 years and visited the periodontology department at the Rajasthan University of health sciences — College of Dental Sciences and Hospital, Jaipur, were selected based on inclusion criteria. The ethical clearance was given by the Ethical Committee and Review Board of the Rajasthan University of Health sciences — College of Dental Sciences and Hospital, Jaipur. After obtaining ethical approval and written informed and verbal consent, the patients agreed to participate in this study. (RUHS-CDS/EC/2023/PG-The/12, dated 30/08/23). This study was conducted from November 2023 to March 2025. This trial was approved and registered at https://ctri.nic.in/ (Registered number: CTRI/2025/02/081311) (**Figure 1**). This study was reported in accordance with the Consolidated Standards of Reporting Trials (CONSORT) guidelines.

**Figure 1:**
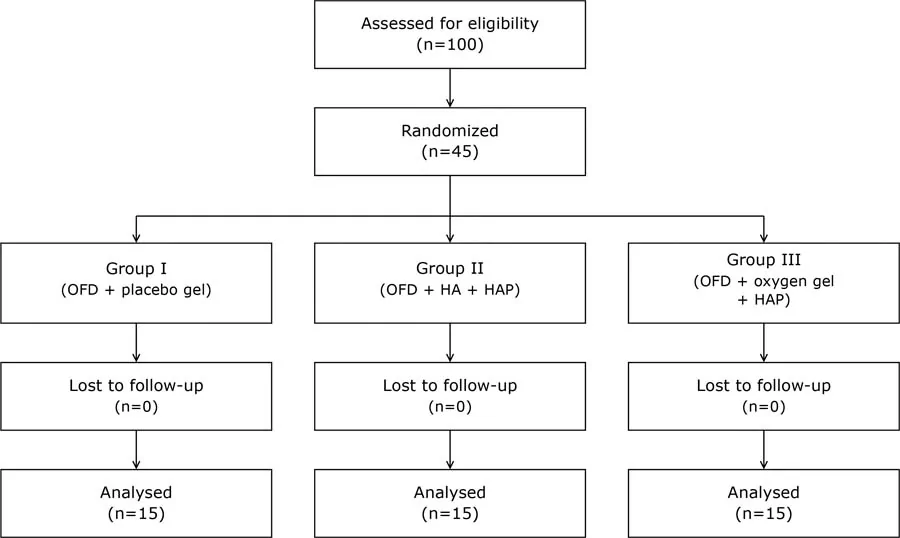
– Consort flow diagram.

### Selection criteria

The inclusion criteria were buccal class II furcation defects in mandibular first molars that are endodontically vital along with a radiolucency in the furcation area according to cone beam computed tomography (CBCT), vertical probing depth (VPD) ≥ 5mm, and horizontal probing depth (HPD) ≥ 3mm following phase I therapy, that is, professional mechanical plaque removal.

Exclusion criteria were patients with systemic diseases, pregnant or lactating women, patients with periodontitis (with stage IV and grade C), tobacco users, and immune-compromised individuals.

The patients were enrolled by the examiner. Sites were then randomly assigned by a computer-generated system to either the open flap debridement (OFD) + placebo group (n=15), OFD+ oxygen gel + HAP group (n=15), or OFD+ HA + HAP group (n=15). (**Figure 1**).

The formula for the minimum sample size is as follows:^21^


$$\frac{n=2\sigma^{2}{\left(Z_{\text{crit }}+Z_{\text{pow }}\right)}^{2}}{D^{2}}$$

in which n is the sample size for each group, σ^2^is the variance of either group (assumed to be equal for both groups), and *D* is the minimal detectable difference between the two means. Z_crit_ and Z_pow_ are the standard normal deviates at a set level of significance and at 1-β power, respectively. A standard normal deviate is the obtention of a standard normal random variable. Z_crit_ equals 1.96 at 5% level of significance for two-sided tests. Z_pow_ totals 0.84 at 80% power and 1.28 at 90% power.

A 2-3-mm thick stent was made with cold cure acrylic. Vertical grooves were made on the stent on its buccal and lingual side using straight fissure bur no. 556 and an airotor hand piece to guide the UNC-15 probe at selected sites. The stent was set to 1/3 on the occlusal/incisal surfaces of the teeth. The measurement was made using a UNC-15 periodontal probe, placing it in the groove made on the stent.

Clinical parameters such as plaque index, gingival index, probing pocket depth (PPD), relative vertical clinical attachment level (RV-CAL), and relative horizontal clinical attachment level (RH-CAL) were recorded at baseline and six months later. A custom acrylic stent with grooves, color-coded periodontal probe (PCP- UNC 15 probe; Hu- Friedy, Chicago, IL, USA) for vertical measurement, and a furcation probe (Nabers furcation probe; Hu- Friedy, USA) for horizontal measurements were used to standardize the clinical parameters.

All CBCT images were taken using Care stream CS9300 imaging system consisting of an X-ray tube, a source collimator, and an amorphous silicon flat-panel detector (CS 3D Imaging v3.5.7, Care stream Health, Inc New York, USA) with a 10×10-cm field of view. Intrabony defect (IBD) distance was measured from the furcation fornix to the base of the defect, being evaluated at baseline and six months later. The percentage bone fill was calculated by the following formula: Percentage bone fill = [(pre-operative furcation depth – post-operative furcation depth) × 100]. This study was double blinded; a single examiner took the baseline and post-operative measurements. One surgeon performed the procedures. Radiographic analysis was performed by a single radiologist. All three gels were packaged identically, and both the patients and the physicians were blinded to treatment allocation.

### GEL USED IN GROUPS 1, 2, and 3

The oxygen-releasing gel in this study is a commercially available gel (Blue^®^m gel) that was developed by Dr. Peter Blijdorp. It contains aqua, alcohol, glycerin, silica, sodium saccharin, sodium perborate, citric acid, PEG-32, sodium gluconate, lactoferrin, xanthan gum, cellulose gum.^22^

The HA gel (0.8%) in this study is a commercially available gel (Gengigel^®^ Prof syringes, Ricerfarma, Milan, Italy). This colorless viscous gel has a characteristic odor, a 6.5± 0.5 pH (at 20°C), and a 1040 ± 0.005 density g/cm^3^ (at 20°C). Its ingredients are water, 7.5% xylitol, 0.8% sodium hyaluronate, and excipients.^23^The placebo gel was formulated by dissolving 2% methylcellulose in triple-distilled water to prepare a methylcellulose gel. The resulting gel was sonicated and sterilized using gamma radiation before use. Full mouth surgeries were performed in the patients after the furcation surgery as results show reduction in overall plaque & gingival scores.

### Surgical Procedure

Consent forms were duly signed by the patients prior to the surgical procedure. All patients were operated under local anesthesia with a solution of 2% lignocaine with 1:80,000 adrenaline. Conventional flap (Kirkland flap) was performed and the furcation area was debrided in all groups. In Group 1, placebo gel was placed; in Group 2, HA and HAP; and in Group 3, oxygen-releasing oral gel and HAP. The flap was sutured to its original position (**Figure 2-4**).

**Figure 2:**
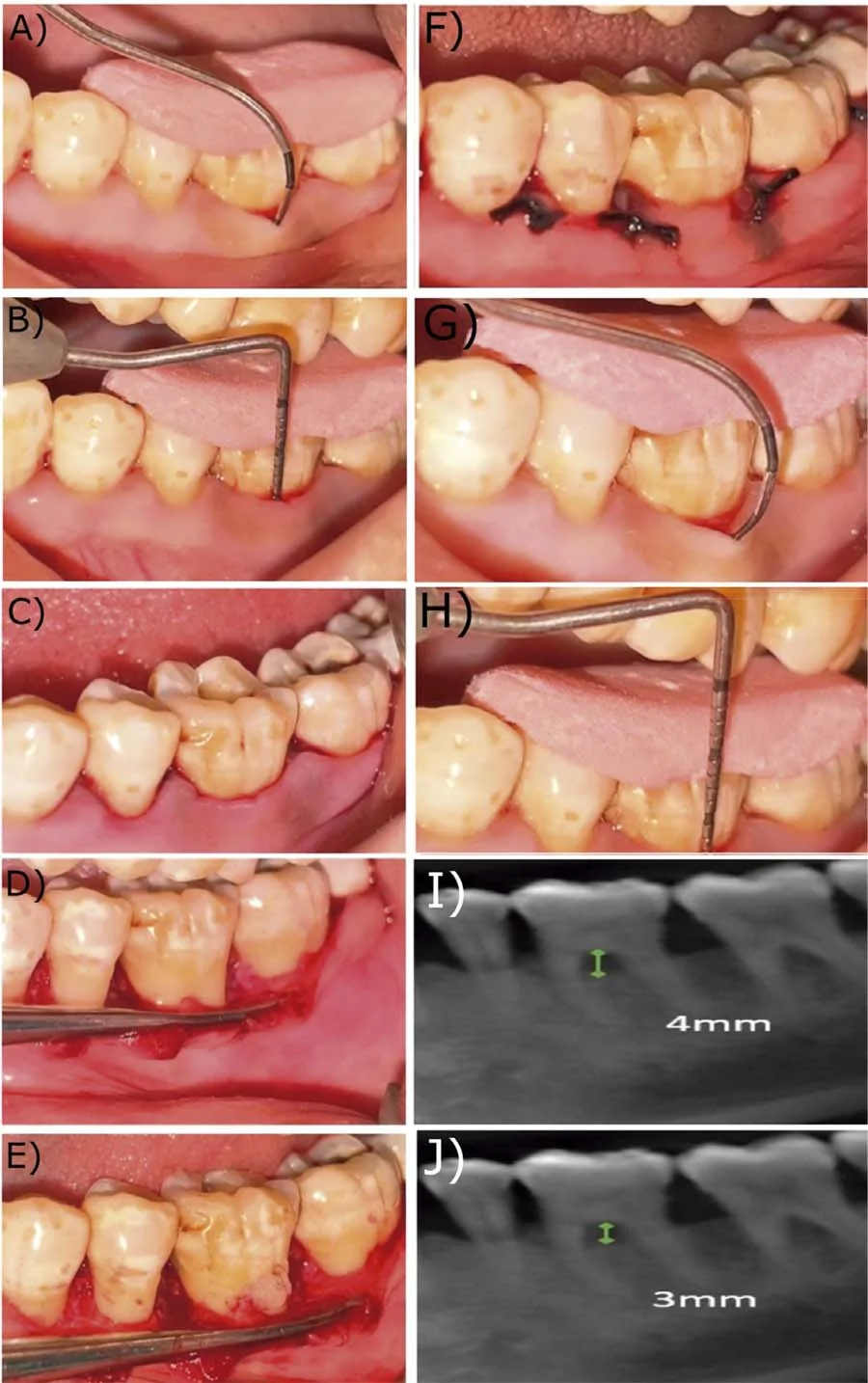
– Group 1 (A) Pre-surgical measurement of HPD of group 1 (B) pre-surgical measurement of VPD of group 1 (C) sulcular incision (D) full thickness flap reflected (E) placebo gel (F) suture placed (G) post-surgical measurement of HPD of group 1 (H) post-surgical measurement of VPD of group 1 (I) pre-operative cross-sectional view of CBCT (J) post-operative cross-sectional view of CBCT.

**Figure 3 f03:**
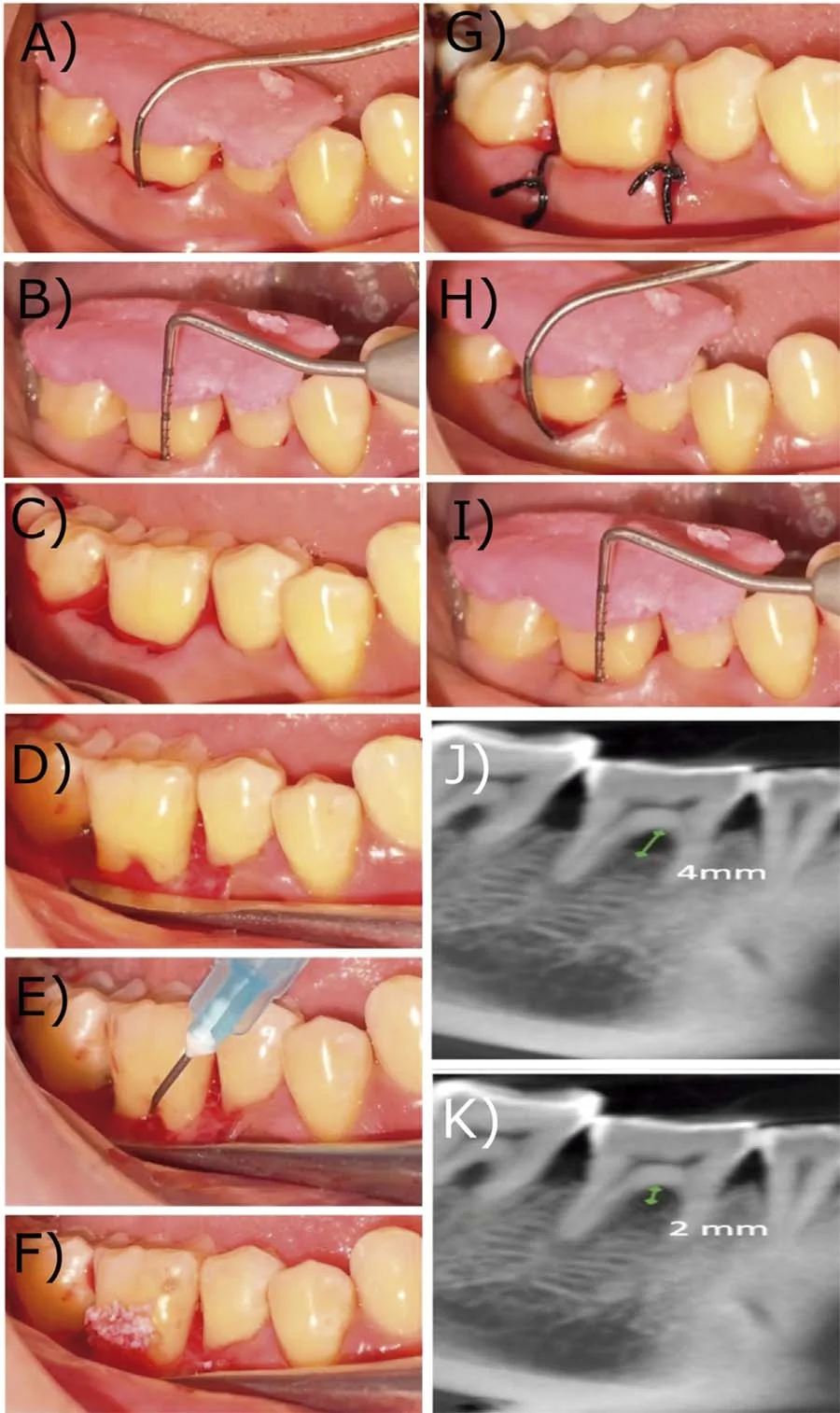
– Group 2 (A) Pre-surgical measurement of HPD of group 2 (B) pre-surgical measurement of VPD of group 2 (C) sulcular incision (D) full thickness flap reflected (E) local delivery of hyaluronic acid gel (F) packing the furcation defect with HAP (G) sutures placed (H) post-surgical measurement of HPD of group 2 (I) post-surgical measurement of VPD of group 2 (J) pre-operative cross-sectional view of CBCT (K) Post-operative cross-sectional view of CBCT.

**Figure 4:**
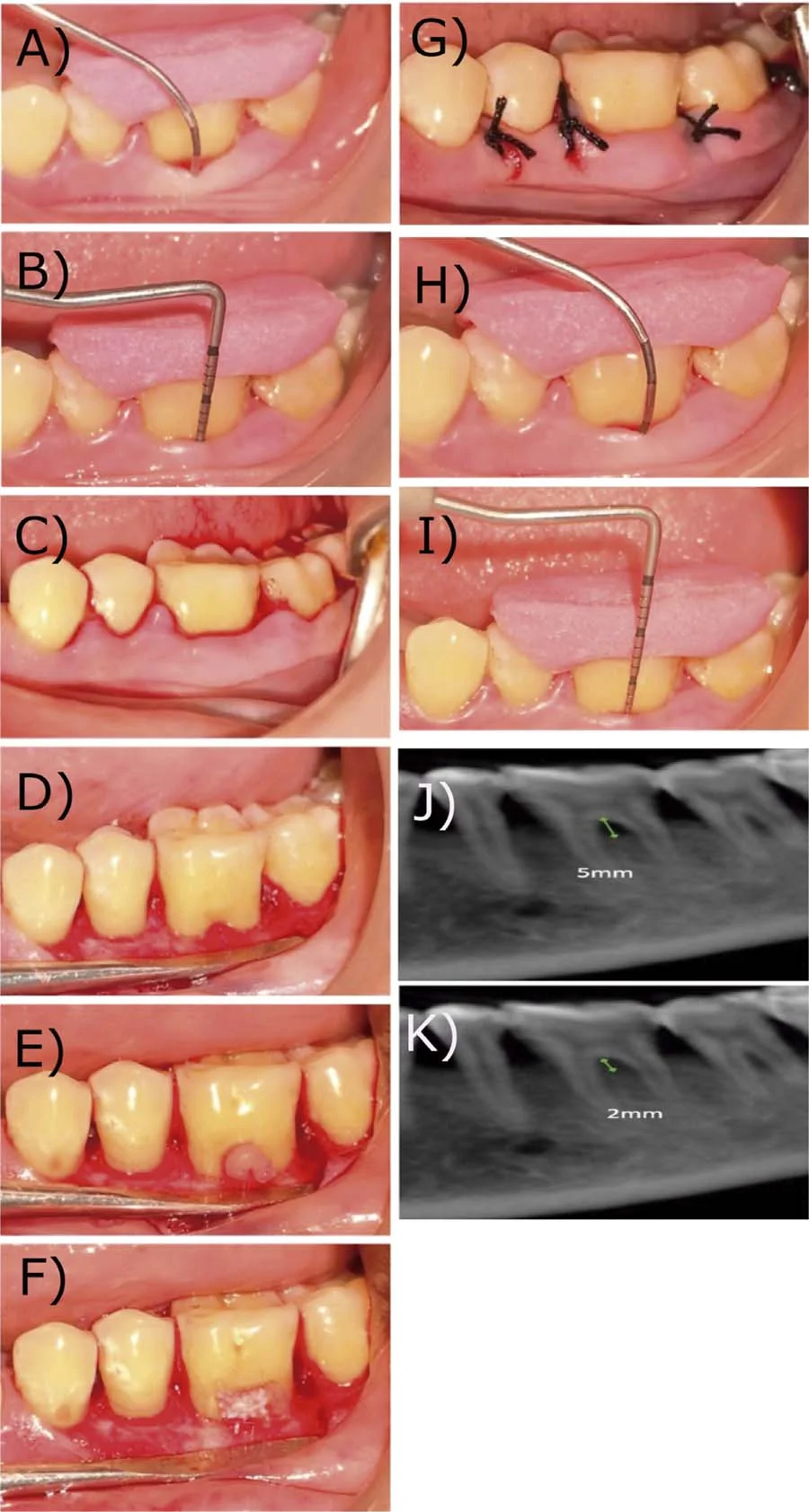
– Group 3 (A) pre-surgical measurement of HPD of group 3 (B) pre-surgical measurement of VPD of group 3 (C) sulcular incision (D) full thickness flap reflected (E) local delivery of oxygen-releasing gel (F) packing the furcation defect with HAP (G) suture placed (H) post-surgical measurement of HPD of group 3 (I) post-surgical measurement of VPD of group 3 (J) pre-operative cross-sectional view of CBCT (K) post-operative cross-sectional view of CBCT.

### Post-Operative Care

Antibiotics (500-mg amoxicillin capsules thrice daily for five days) and analgesics (400-mg ibuprofen tablets thrice daily for five days) were advised since systemic antibiotics can improve attachment levels when used as adjuncts to root instrumentation.^24^ Chlorhexidine mouthwash (0.2%) twice daily was also prescribed for two weeks. The interrupted direct loop sutures and periodontal dressings were removed one week after surgery. Patients were on maintenance phase every three-six months (which included prophylaxis). Oral hygiene guidance was emphasized at every visit. Evaluation of clinical parameters was performed at the end of post-operative six months. CBCT was used to assess hard tissue assessment (bony defect). Bone defect measurements were re-evaluated at six months (**Figure 2-4**).

### Statistical Analysis

The data in this study was input onto Microsoft Excel 2007 and analyzed on SPSS, version 23.0. Descriptive statistics included means and standard deviations. Intergroup comparisons were performed via one way analysis of variance to find the difference between groups. Intragroup comparisons between time intervals were performed by the paired t-test. Significance level was set at 5% in this study. The Shapiro–Wilk test was used to investigate data distribution. Variable homogeneity was explored by Levene’s test.

For the statistical tests, p<0.05 was considered as statistically significant under α 5% error and a 20% β error, rendering an 80% power for this study.

## Results

All 45 patients completed this study with neither withdrawals nor reported adverse effects. Healing was uneventful without post-procedure complications.

Out of 45 participants, Group 1 had 15 participants with a mean age of 30.60 years, Group 2 had 15 participants with a mean age of 27.87 years, and Group 3 had 15 participants with a mean age of 32.47 years. Of the 45 participants, 21 were women and 24 were men (**Table 1**).

**Table 1:** intergroup comparison of age and gender distribution.

Groups	Gender	Age	Mean age (years)
1	male	10	30.60
	female	5	
2	male	8	27.87
	female	7	
3	male	6	32.47
	female	9	
**Total**		**45**	

The frequency of age and gender values a statistically but non-significantly differed between the groups (p>0.05).

The intergroup comparison found non-significant differences in plaque and gingival indices at baseline and at the six-month follow-up between the groups (which remained statistically non-significant — p>0.05), indicating that all groups showed comparable biofilm reduction and gingival health improvement over time.

The intragroup comparison of PPD between baseline and six months showed a significant reduction in all three groups (p=0.001) for all comparisons (**Table 2**). At baseline, the intergroup comparison of PPD were non-significant. After six months, intergroup comparison of mean difference between Groups 3 and 1 (p=0.004) and Groups 3 and 2 (p=0.046) were significant in Groups 3, whereas Groups 1 and Group 2 showed a statistically non-significant difference in PPD (p=0.192) (**Table 5**).

**Table 2:** Intragroup comparison of PPD between baseline and six months in all the groups.

Group	Baseline	6 Months	Mean Change	P value	Significance
Group 1	6.667±1.047	5.200±1.207	1.467±0.834	0.001	**Significant**
Group 2	6.533±1,060	4.733±0.884	1.800±0.676	0.001	**Significant**
Group 3	6.600±0.828	4.133±0.743	2.467±0.516	0.001	**Significant**

**Table 5:** Intergroup comparison of mean difference clinical parameters at six months.

Parameters	Intergroup comparison		Mean Difference	P value	Significance
PI	Group 1	Group 2	0.200	0.413	Non-Significant
	Group 1	Group 3	0.267	0.276	Non-Significant
	Group 2	Group 3	0.067	0.784	Non-Significant
**GI**	Group 1	Group 2	0.666	0.071	Non-Significant
	Group 1	Group 3	0.733	0.051	Non-Significant
	Group 2	Group 3	0.067	0.743	Non-Significant
**PPD**	Group 1	Group 2	0.467	0.192	Non-Significant
	Group 1	Group 3	**1.067**	**0.004**	**Significant**
	Group 2	Group 3	**0.600**	**0.046**	**Significant**
**RVCAL**	Group 1	Group 2	0.267	0.241	Non-Significant
	Group 1	Group 3	**1.000**	**0.000**	**Significant**
	Group 2	Group 3	**0.733**	**0.002**	**Significant**
**RHCAL**	Group 1	Group 2	0.400	0.081	Non-Significant
	Group 1	Group 3	**1.467**	**0.000**	**Significant**
	Group 2	Group 3	**1.067**	**0.000**	**Significant**

The intragroup comparison of RV-CAL between baseline and six months showed statistically significant reductions in all three groups (**Table 3**). The intergroup comparison of the mean difference in RV-CAL at baseline were non-significant (p>0.05). At six months, the post hoc analysis of Group 1 vs. 2 was non-significant (p=0.241), whereas that of Group 1 vs Group 3 (p<0.001) and of Group 2 vs Group 3 (p=0.002) were significant, indicating greater attachment gain in Group 3. (**Table 5**)

**Table 3:** Intragroup comparison of relative vertical clinical attachment level between baseline and six months in all the groups.

Group	Baseline	6 Months	Mean Change	P value	Significance
Group 1	5.200±0.775	4.267±0.594	0.933±0.704	0.001	**Significant**
Group 2	5.133±0.743	4.000±0.655	1.133±0.516	0.001	**Significant**
Group 3	4.933±0.704	3.267±0.594	1.667±0.488	0.001	**Significant**

The intragroup comparison of RH-CAL between baseline and six months showed a statistically significant reduction in all three groups (p=0.001) (**Table 4**) The intergroup comparison of mean difference in RH-CAL at baseline were non-significant a (p>0.05). At six months, the post hoc analysis of Group 1 vs. Group 2 were non-significant (p=0.081), whereas that Group 1 vs Group 3 (p<0.001) and of Group 2 vs Group 3 (p<0.001) were significant (**Table 5**).

**Table 4:** Intragroup comparison of RH-CAL between baseline and six months in all the groups.

Group	Baseline	6 Months	Mean Change	P value	Significance
Group 1	5.467±0.516	4.600±0.632	0.867±1.060	0.001	**Significant**
Group 2	5.467±0.516	4.200±0.775	1.267±1.033	0.001	**Significant**
Group 3	5.733±0.458	3.133±0.353	2.600±0.737	0.001	**Significant**

The intergroup comparison of IBD mean values at baseline were non-significant. At six months, the mean values of IBD showed a significant difference between Groups 3 and 1 (p=0.001) and between Groups 3 and 2 (p=0.001), whereas the difference between Groups 1 and Group 2 was non-significant (**Figure 5**).

**Figure 5:**
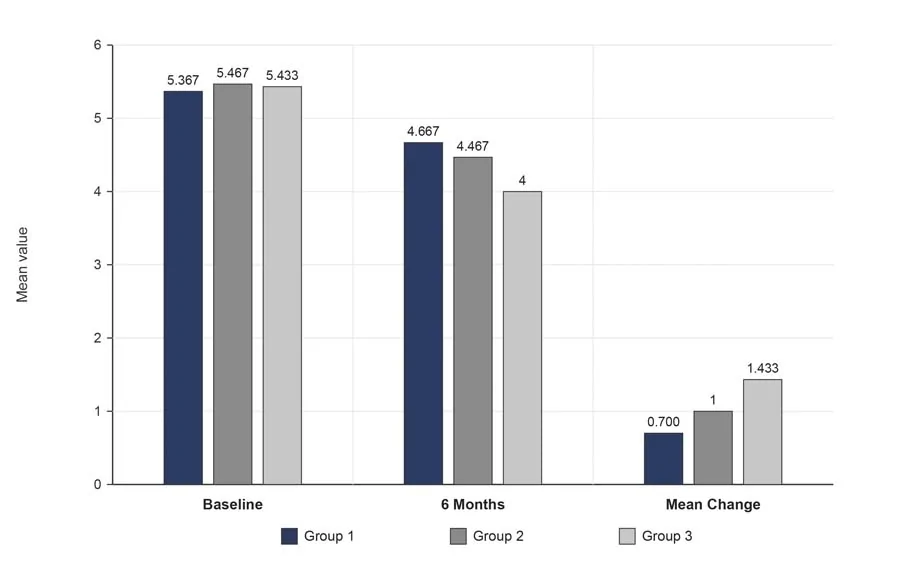
Intergroup comparison of IBD between the groups.

Regarding bone fill percentage, Group 3 (50.93%±4.62) showed higher values than Groups 2 (46.87%±4.71) and 1 (34.69%±12.14) (**Figure 6**).

**Figure 6:**
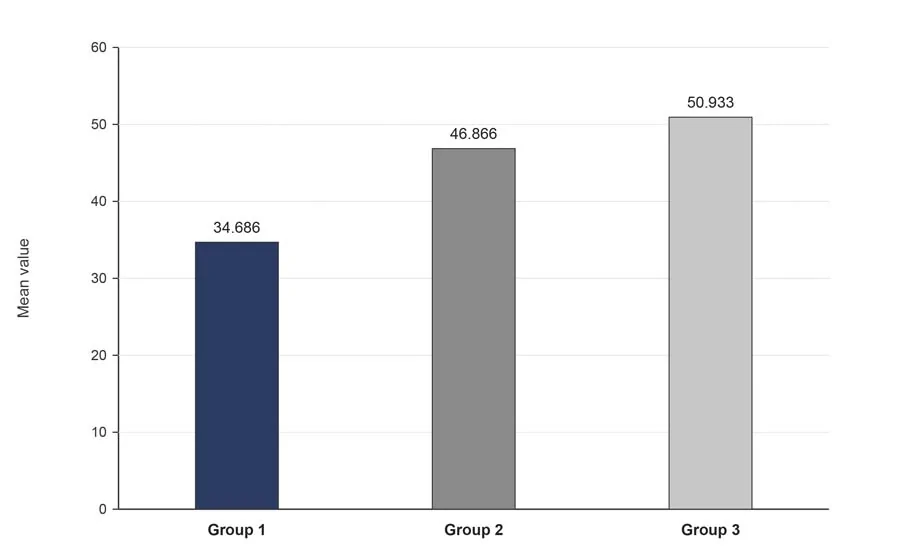
Intergroup comparison of % bone fill between the groups.

## Discussion

Numerous investigations have shown that the furcation defect of multirooted teeth can be improved by the local delivery of various pharmaceutical agents.^25,26^ Evidence suggests a combination approach. Moreover, adding biological mediators provides the best treatment of Class II defects.

Paradigm shift in periodontal research has led researchers to seek new biomaterials to enhance and accelerate individuals’ intrinsic healing potential. Advances in the understanding of inflammatory mechanisms and wound healing processes associated with periodontal diseases have led researchers to study the potential of numerous extracellular matrix components as promoters of periodontal healing and regeneration. One such component is oxygen-releasing gel. A crucial nutrient for cellular metabolism, particularly energy synthesis, is oxygen. This substance is engaged in several processes, including collagen formation, angiogenesis, re-epithelialization, and oxidative death of bacteria.^27,28^

Clinical parameters such as plaque index, gingival index, PPD, RV-CAL, and RH-CAL were evaluated at baseline and six months after surgery in addition to the radiographic assessment of defect fill. CBCT analysis is used to radiographically assess bony defect fill. The advantages of CBCT analysis include its rapid scan time (30 seconds) and less radiation exposure than panoramic radiographs.

The improvement in plaque index and gingival scores in this study may be attributed to the fact that the oral hygiene was satisfactorily maintained in all subjects. This suggests that regular reinforcement of the oral hygiene instructions was beneficial and helpful in creating patient awareness regarding the need for oral hygiene. The Hawthorne effect might have played an important role regarding the maintenance of oral hygiene, as in McCarney, et al.^29^ (2007).

After six months, the intergroup comparison of PPD between Groups 3 and 1 (p=0.004) and Groups 3 and 2 (p=0.046) were significant. This suggests that Groups 3 showed a greater reduction in PPD than the other Groups. Similar results were shown in Singh, et al.^30^ (2024). The decline in PPD in Group 3 is due to the combined effect of oxygen-releasing gel and HAP. The former has anti-inflammatory, tissue-forming effects and encourages fibroblast and keratinocyte migration and proliferation, re-epithelialization, and novice granulation tissue formation locally and systemically.^31^HAP shows mechanical properties closely resembling those of natural human bone, making it an effective bone-mimicking material. This property renders HAP a promising candidate for applications in guided tissue regeneration.^32^No Group involved furcation treatment with HAP only in this study—as in Figliuzzi, et al.^32^ (2016), a similar study to this one.

The intergroup comparison of RV-CAL and RH-CAL showed significant furcation area vertical and horizontal attachment gain in Group 3 (in which oxygen-releasing gel and HAP bone graft were placed). Oxygen therapy increases vascular endothelial growth factor expression, which encourages progenitor cells to differentiate, leading to neovascularization and regeneration. HAP acts as a framework for new cells formation. A similar study, Agarwal, et al.^33^ (2024), found greater clinical attachment level in its test group (oxygen-releasing gel + scaling and root planing) than in the control group (scaling and root planing alone) because of active oxygen release by the gel leading to progressive tissue healing, showing a synergistic effect that helps to kill anaerobic bacteria.

The intergroup comparison found the highest IBD reduction and bone fill percentage in Group (p-value = 0.001) than in the other Groups. Local lactoferrin administration significantly enhanced bone regeneration. Lactoferrin, a main component of oxygen-releasing gel has been found to be a potential anabolic factor for bone growth. It stimulates the proliferation and differentiation of bone-forming cells (osteoblasts) in a dose-dependent manner while significantly inhibiting the production of bone-resorbing cells. Hence, this could explain the higher bone fill percentage in Group 3 (OFD + oxygen gel + HAP). Similarly, in Palwankar, Gupta, and Pandey^34^ (2020), nano-crystalline hydroxyapatite and bioactive calcium phosphosilicate grafts showed greater bone fill percentages when treating class II furcation defects. This is due to the silica-rich layer provided by hydroxycarbonate-apatite precipitation and crystallization, increasing osteoblast cell proliferation and enabling rapid bone formation. This study also found a significant bone fill percentage in Group 3, which received hydroxyapatite and oxygen-releasing oral gel together.

Mattei, et al.^27^ (2021) showed a notable decrease in bone defect depth under an oxygen-releasing formulation. The osteoblastic impact caused the outcomes. It also released lactoferrin and hydrogen peroxide, which have antimicrobial and bone-cell-stimulating properties, respectively. The limitations of the study refer to its small sample size. Long-term analysis of results is required to determine treatment outcome stability. The regeneration clinical results in this study require support by histological analysis. Future studies with longer follow-up periods and histological analyses are required to further evaluate the long-term outcomes and regenerative potential of the oxygen-releasing gel treatment.

## Conclusion

Within the limitations of this study, its results suggest statistically significant mean relative attachment level gain and mean probing pocket depth reduction in all three Groups from baseline and six post-operative months. The intergroup comparison found highly significant results at each time interval. The intergroup comparison found significantly greater mean RH-CAL & RV-CAL gain and PPD reduction in OFD + oxygen-releasing gel + hydroxyapatite bone graft (Group 3) than in OFD + 0.8% HA gel + hydroxyapatite bone graft (Group 2) and OFD + placebo gel (Group 1). The results suggest that combining oxygen-releasing gel and hydroxyapatite in surgical periodontal therapy has shown superior regenerative potential. These ﬁndings may give new insight into the use of oxygen-releasing gel in periodontal regenerative techniques.
